# Safety and Efficacy of Tirofiban for Acute Ischemic Stroke Patients With Large Artery Atherosclerosis Stroke Etiology Undergoing Endovascular Therapy

**DOI:** 10.3389/fneur.2021.630301

**Published:** 2021-02-11

**Authors:** Xiaochuan Huo, Raynald, Anxin Wang, Dapeng Mo, Feng Gao, Ning Ma, Yilong Wang, Yongjun Wang, Zhongrong Miao

**Affiliations:** ^1^Neurointervention Center, Beijing Tiantan Hospital, Capital Medical University, Beijing, China; ^2^Department of Neurology, Beijing Tiantan Hospital, Capital Medical University, Beijing, China; ^3^China National Clinical Research Center for Neurological Diseases, Beijing, China; ^4^Center of Stroke, Beijing Institute for Brain Disorders, Beijing, China

**Keywords:** tirofiban, endovascular therapy, acute ischemic stroke, large artery atherosclerosis, safety and efficacy, clinical outcome

## Abstract

**Objective:** To investigate the safety and efficacy of tirofiban in acute ischemic stroke (AIS) patients with large artery atherosclerosis (LAA) stroke etiology receiving endovascular therapy (EVT).

**Methods:** In this multi-center prospective study, patients who were considered to have an indication received a low dose intra-arterial bolus (0.25–1 mg) of tirofiban. The safety and efficacy outcomes at 90-day follow-ups included symptomatic intracranial hemorrhage (sICH), recanalization rate, functional outcome, and mortality.

**Results:** Among the 649 AIS patients with LAA, those in the tirofiban group (*n* = 244) showed higher systolic blood pressure (BP) and NIHSS score on admission, puncture-to-recanalization time, lower frequency of intravenous thrombolysis and intra-arterial thrombolysis, higher frequency of antiplatelet, heparinization, mechanical stent retrieval, aspiration, balloon angioplasty, and more retrieval times compared with those in the non-tirofiban group (*n* = 405) (all *P* < *0.05*). Tirofiban was found to be associated with superior clinical outcomes in anterior circulation stroke and major stroke patients [adjusted odds ratio (OR) = 2.163, 95% confidence interval (CI) = 1.130–4.140, *P* = *0.02* and adjusted OR = 2.361, 95% CI = 1.326–4.202, *P* = *0.004*, respectively] and a lower risk of mortality at 90-day follow-ups (adjusted OR = 0.159, 95% CI = 0.042–0.599, *P* = *0.007* and adjusted OR = 0.252, 95% CI = 0.103–0.621, *P* = *0.003*, respectively). There was no significant difference in sICH between the two groups.

**Conclusions:** Tirofiban in AIS patients with LAA undergoing EVT is safe and may benefit the functional outcomes in anterior circulation and major stroke patients and showed a trend for reduced mortality.

## Introduction

The non-peptide platelet GP IIb/IIIa receptor inhibitor, tirofiban, has been increasingly applied as a rescue therapy, by either intra-arterial or intravenous route during endovascular treatment (EVT) (1–8). Tirofiban can selectively and efficiently block the final pathway of platelet aggregation and subsequent thrombus formation in atherosclerotic lesions (9, 10). Recent metaanalysis studies have reported that the safety profile and efficacy of tirofiban may make it a potential choice for treatment in patients with acute ischemic stroke (AIS) (11–14). It has also been reported to be more feasible and effective in AIS patients with large artery atherosclerosis (LAA) compared to those with cardioembolic stroke etiology (15, 16). However, the treatment results were inconsistent (1, 17, 18) and a study reported an increased risk of symptomatic intracranial hemorrhage (sICH) and a poor outcome in patients treated with tirofiban during mechanical thrombectomy (19). Moreover, to the best of our knowledge, there are no reports on which stratified population may benefit the most from rescue tirofiban therapy.

To address this issue, we explored the safety and efficacy of rescue tirofiban treatment in AIS patients with LAA stroke etiology and evaluated which stratified population gained the most benefit from rescue tirofiban in a large multi-center cohort study in China.

## Methods

### Patient Selection and Data Collection

This multi-center nationwide prospective study of an Acute Ischemic Stroke Cooperation group in the Endovascular Treatment (ANGEL) registry recruited 917 Chinese patients with AIS to evaluate EVT delivery and improve EVT. The study protocol was similar to our previous research (20). The present study was approved by the ethics committee at each participating center, and informed consent was obtained from all participants prior to commencing the study.

Patient's baseline data, such as age, gender, systolic blood pressure (SBP), the National Institutes of Health Stroke Scale (NIHSS) score, Alberta Stroke Program Early CT Score (ASPECTS), time intervals [onset-to-door (OTD), door-to-puncture (DTP), puncture-to-recanalization (PTR), onset-to-puncture (OTP), and onset-to-recanalization (OTR)], were recorded within 24 h after admission. Vascular risk factors included atrial fibrillation, diabetes mellitus, history of previous stroke, hypertension, smoking, and drinking. The data related to the peri-procedural anti-thrombotic and anticoagulation therapies, such as administration of antiplatelets, bridging intravenous thrombolysis (IVT), and heparin, were recorded as along with the procedural techniques.

AIS patients undergoing EVT were divided into tirofiban and non-tirofiban groups. All EVT procedures were performed by neurointerventionalists with extensive experience in neurovascular intervention.

### Dose and Indication of Rescue Tirofiban

Rescue tirofiban with low-dose intra-arterial bolus (0.25–1 mg) is suggested when there are the following indications: (1) severe residual stenosis or instant re-occlusion requiring emergency stenting or balloon angioplasty; (2) stent retrieval times > 3 passes for presumed vascular endothelial injury or instant re-occlusion; and (3) severe degree of *in situ* atherosclerosis with a tendency to early re-occlusion. Low dose rescue tirofiban followed by intravenous continuous infusion (0.1 μg/kg/min) for 12–24 h is suggested when there is no indication of post-operative intracranial hemorrhage following a CT examination.

### Clinical Efficacy and Safety Outcomes

SICH, which was defined by the European Cooperative Acute Stroke Study III (ECASS-III) trial as evidence of hemorrhage on a CT or MRI, was considered a primary safety endpoint. The primary efficacy endpoints were the functional independence (mRS 0-2) and mortality at 90 day follow-ups. A successful recanalization, which was defined as modified Thrombolysis in Cerebral Infarction (mTICI), is considered the secondary efficacy endpoint in the present study.

### Statistical Analysis

The baseline characteristics of patients were compared between the tirofiban and non-tirofiban groups. The χ^2^ test or Kruskal-Wallis test was used to compare the baseline characteristics and safety and efficacy outcomes at 90 days between the tirofiban and non-tirofiban groups. The logistic regression model was used to evaluate the odds ratios (OR)/hazard ratio (HR) with a 95% confidence interval (CI) of safety and efficacy endpoints (sICH), mTICI grade 2b-3, complete reperfusion (mTICI 3), functional independence (mRS 0-2), and mortality with or without use of tirofiban. The multivariate models were adjusted for some potential confounders with *P* < *0.05* in univariate analysis, which included SBP, NIHSS, atrial fibrillation, smoking history, anterior and posterior circulation, occlusion of the M2 or M3 segment of the middle cerebral artery (MCA) M2/3 segment, vertebral artery (VA), basilar artery (BA), posterior cerebral artery (PCA), antiplatelet, bridging IVT during EVT, general anesthesia, MT stent retrieval and aspiration, balloon angioplasty and intra-arterial thrombolysis, and retrieval times. A *P-value* < *0.05* was considered statistically significant. All statistical analyses were conducted using SPSS 20.0 software (IBM, Armonk, NY, USA).

## Results

### Baseline Characteristics of Patients

**Figure 1:**
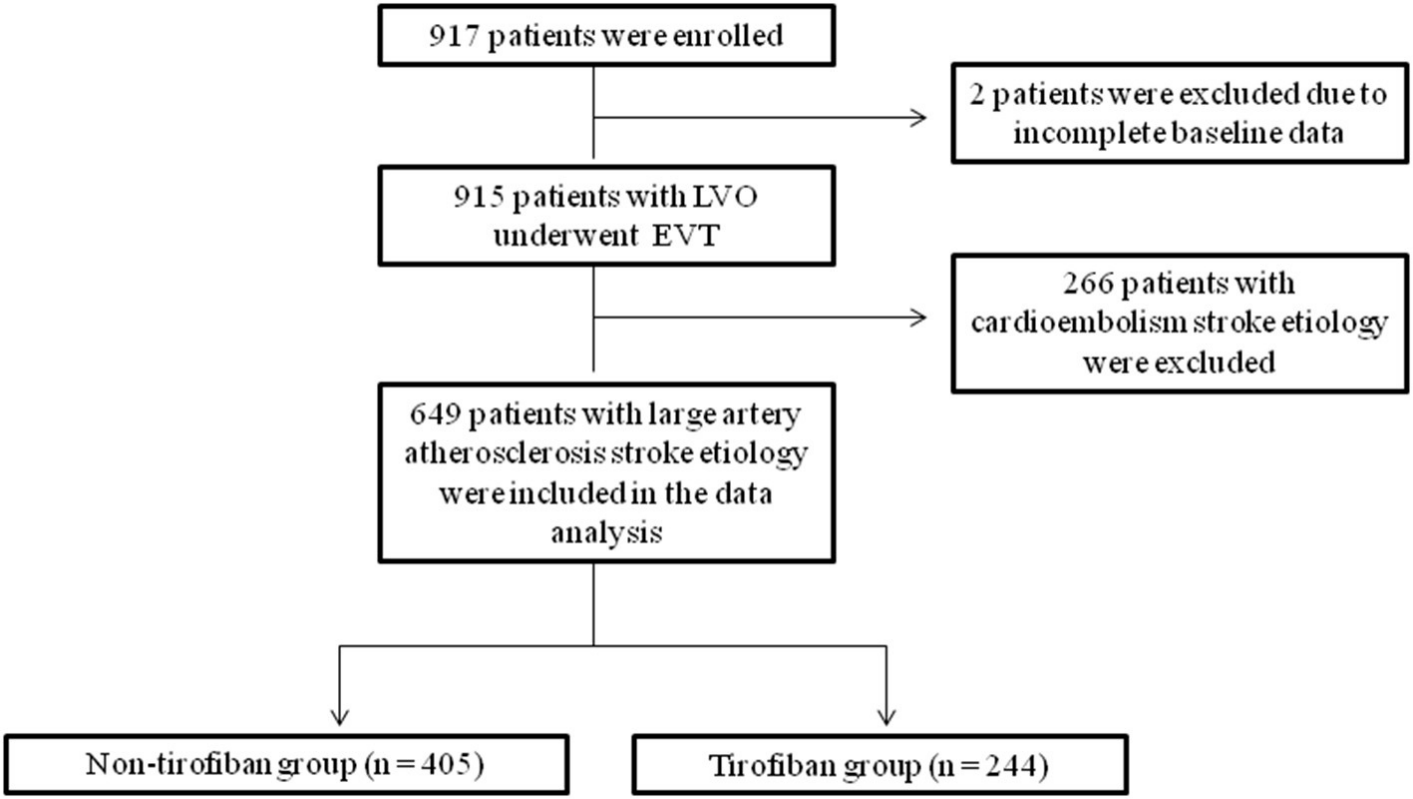
Flow chart showing patient selection.

Two of the 917 patients were excluded from the data analysis due to missing baseline data. Subsequently, 266 patients with embolic stroke etiology were also excluded. Finally, 649 patients with large vessel atherosclerosis who underwent EVT with or without receiving tirofiban were analyzed (Figure 1).

As shown in Table 1, the median age of patients was 63 (55–71) years, 464 (71.5%) patients were male, and 244 (37.6%) had received tirofiban. In the tirofiban group, SBP and NIHSS on admission were relatively higher and smoking history was more frequent, while atrial fibrillation was less obvious than those in the non-tirofiban group (all *P* < *0.05*). Rescue tirofiban was used more in the posterior circulation (particularly VA, BA, and PCA), but less in the anterior circulation group (particularly MCI M23 segment). In the tirofiban group, general anesthesia, stent retrieval, MT aspiration, and balloon angioplasty were more frequently performed as compared to the non-tirofiban group (45.5 vs. 26.2%, *P* < *0.001*), (76.2 vs. 59.8%), *P* < *0.001*), (10.2 vs. 2.7%), *P* < *0.001*), and (17.6 vs. 10.4%), *P* = *0.008*)), respectively. Moreover, anti-platelet therapy was administered more in the tirofiban group (38.1 vs. 13.6%), *P* < *0.001*). Meanwhile, the proportions of bridging IVT and intra-arterial thrombolysis were less in the tirofiban group compared to the non-tirofiban group (20.1 vs. 30.9%, *P* = *0.003*) and (11.5 vs. 30.6%, *P* < *0.001*).

**Table 1:** Patient's Baseline and procedural characteristics.

**Variables**	**Total (*n* = 649)**	**Non-tirofiban (*n* = 405)**	**Tirofiban (*n* = 244)**	***P*-value**
Age, mean ± SD	63 (55–71)	63 (54–71)	64 (55–70.75)	0.784
Male	464 (71.5)	282 (69.6)	182 (74.6)	0.175
SBP, mean + SD	148 (133–162)	147 (130–160)	150 (138–168.75)	**0.037**
Admission NIHSS, median (IQR)	13 (8–18)	13 (7–17.5)	14 (10–20.75)	**0.005**
ASPECTS (AC only)	8 (7–8)	8 (7–8)	8 (7–8)	0.959
Vascular risk factors				
Atrial Fibrillation	55 (8.5)	43 (10.6)	12 (4.9)	**0.012**
Diabetes Mellitus	107 (16.5)	65 (16)	42 (17.2)	0.699
Previous stroke	70 (10.8)	40 (9.9)	30 (12.3)	0.336
Hypertension	376 (57.9)	224 (55.3)	152 (62.3)	0.081
Smoking	238 (36.7)	132 (32.6)	106 (43.4)	**0.005**
Drinking	111 (17.1)	65 (16)	46 (18.9)	0.358
Anterior circulation	488 (75.2)	324 (80)	164 (67.2)	** <0.001**
Posterior circulation	161 (24.8)	81 (20)	80 (32.8)	** <0.001**
Occlusion sites				
ICA	209 (32.2)	137 (33.8)	72 (29.5)	0.254
M1	225 (34.7)	147 (36.3)	78 (32)	0.262
M2/3	50 (7.7)	38 (9.4)	12 (4.9)	**0.039**
ACA	4 (0.6)	2 (0.5)	2 (0.8)	1
VA	78 (12)	39 (9.6)	39 (16)	**0.016**
BA	68 (10.5)	28 (6.9)	40 (16.4)	** <0.001**
PCA	15 (2.3)	14 (3.5)	1 (0.4)	**0.026**
OTD time, median (IQR), min	180 (110–300)	180 (102–300)	203.5 (120–313.5)	0.076
DTP time, median (IQR), min	116 (74–167.5)	119(80–168.5)	110 (62.25–167.25)	0.123
PTR time, median (IQR), min	80 (55–115)	119 (80–168.5)	110 (62.25–167.25)	0.095
OTP time, median (IQR), min	330 (225–463.5)	320 (223.5–453.5)	340 (233.5–490)	0.239
OTR time, median (IQR), min	420 (320–576)	410 (316.25–550)	438.5 (323.75–629.25)	0.103
Antitrombotic and anticoagulation				
Antiplatelet	148 (22.8)	55 (13.6)	93 (38.1)	** <0.001**
Bridging IVT	174 (26.8)	125 (30.9)	49 (20.1)	**0.003**
Heparin during EVT	242 (37.3)	141 (34.8)	101 (41.4)	0.093
Procedural characteristics				
General anesthesia	217 (33.4)	106 (26.2)	111 (45.5)	** <0.001**
Stent retrieval	428 (65.9)	242 (59.8)	186 (76.2)	** <0.001**
Aspiration	36 (5.5)	11 (2.7)	25 (10.2)	** <0.001**
Intra-arterial thrombolysis	152 (23.4)	124 (30.6)	28 (11.5)	** <0.001**
Balloon angioplasty	85 (13.1)	42 (10.4)	43 (17.6)	** <0.001**
Stent angioplasty	126 (19.4)	70 (17.3)	56 (23)	0.077
retrieval times, median (IQR)	1 (0–1)	1 (0–1)	1 (1–1)	**0.002**

*SD, standard deviation; SBP, systolic blood pressure; IQR, interquartile range; NIHSS, National Institutes of Health Stroke Scale score; ASPECTS, Alberta Stroke Program Early CT score; ICA, internal carotid artery; M1, middle cerebral artery M1 segment; M2/3, middle cerebral artery M2/3 segment; ACA, anterior cerebral artery; OTD, onset-to-door; DTP, door-to-puncture; PTR, puncture-to-recanalization; OTP, onset-to-puncture; OTR, onset-to-recanalization; IVT, intravenous thrombolysis; EVT, endovascular treatment. Bold values indicates statistical significance*.

There was no significant difference in age, gender, other vascular risk factors (diabetes mellitus, previous stroke, hypertension, and drinking), other occlusion sites (ICA, MCA M1, or ACA), time workflow (OTD, DTP, PTR, OTP, and OTR), heparinization during EVT, and stent angioplasty between the tirofiban and non-tirofiban groups (all *P* > *0.05*).

### Safety and Efficacy Outcomes

The safety and efficacy outcomes are shown in Tables 2, 3, 4. Overall, 27 (4.2%) patients developed sICH within 24 h post-EVT, and no significant difference was noted in the sICH incidence between the tirofiban group and the non-tirofiban group (*P* > *0.05*). Tirofiban was not correlated with the incidence of sICH (adjusted HR 0.998; 95% CI 0.021–46.825; *P* = *0.999*) even after adjusting for some potential confounders. Similar results were demonstrated when the population was stratified into anterior/posterior circulation and minor (NIHSS 0–5)/major (NIHSS > 5) stroke (all *P* > *0.05*).

**Table 2:** Safety and efficacy outcomes grouped by tirofiban in LAA patients.

**Variables**	**Total (*n* = 649)**	**Non-tirofiban (*n* = 405)**	**Tirofiban (*n* = 244)**	**OR/HR**	***P*-value**	**adjusted OR/HR**	***P*-value**
Safety outcome							
sICH	27 (4.2)	16 (4)	11 (4.5)	1.148 (0.524–2.516)	0.731	0.998 (0.021–46.825	0.999
Recanalization							
mTICI 2b/3	605 (93.2)	377 (93.1)	228 (93.4)	1.058 (0.56–1.999)	0.861	0.308 (0.104–0.911)	**0.033**
Functional outcome at 90-days							
mRS 0–1	295 (45.5)	182 (44.9)	113 (46.3)	1.057 (0.768–1.454)	0.734	1.819 (1.064–3.110)	**0.029**
mRS 0–2	364 (56.1)	227 (56)	137 (56.1)	1.004 (0.729–1.383)	0.981	1.849 (1.065–3.212)	**0.029**
mRS 6	87 (13.4)	59 (14.6)	28 (11.5)	0.76 (0.470–1.230)	0.264	0.2 (0.079–0.507)	**0.001**

*sICH, symptomatic intracranial hemorrhage; aICH, asymptomatic intracranial hemorrhage; mTICI, modified treatment in cerebral infarction; mRS, modified rankin score; OR, odds ratio; HR, hazard ratio*.

*adjusted for SBP, NIHSS, atrial fibrillation, smoking, anterior circulation, posterior circulation, MCA M23 segment, VA, BA, PCA, antiplatelet, Intravenous thrombolysis, general anesthesia, MT stent retrieval, MT aspiration, intra-arterial thrombolysis, balloon angioplasty and retrieval times. Bold values indicates statistical significance*.

**Table 3:** Safety and efficacy outcomes grouped by tirofiban in LAA patients stratified according to anterior and posterior circulation stroke.

**Anterior Circulation**							
**Variables**	**Total (*****n*** **= 488)**	**Non-tirofiban (*****n*** **= 324)**	**Tirofiban (*****n*** **= 164)**	**OR/HR**	***P*****-value**	**adjusted OR/HR**	***P*****-value**
Safety outcome							
sICH	22 (4.5)	14 (4.3)	8 (4.9)	1.136 (0.466–2.764)	0.779	3.52 × 10^10^ (0)	0.997
Recanalization							
mTICI 2b/3	456 (93.4)	302 (93.2)	154 (93.9)	1.122 (0.518–2.428)	0.77	0.343 (0.053–2.200)	0.259
Functional outcome at 90-days							
mRS 0-1	216 (44.3)	135 (41.7)	81 (49.4)	1.366 (0.937–1.993)	0.105	2.163 (1.130–4.140)	**0.02**
mRS 0-2	272 (55.7)	174 (53.7)	98 (59.8)	1.28 (0.875–1.873)	0.204	1.845 (0.946–3.598)	0.072
mRS 6	53 (10.9)	40 (12.3)	13 (7.9)	0.611 (0.317–1.178)	0.141	0.159 (0.042–0.599)	**0.007**
**Posterior Circulation**							
**Variables**	**Total (*****n*** **= 161)**	**Non-tirofiban (*****n*** **= 81)**	**Tirofiban (*****n*** **= 80)**	**OR/HR**	***P*****-value**	**adjusted OR/HR**	***P*****-value**
Safety outcome							
sICH	5 (3.1)	2 (2.5)	3 (3.8)	1.539 (0.250–9.465)	0.642	2.27 × 10^20^ (0)	0.993
Recanalization							
mTICI 2b/3	149 (92.5)	75 (92.6)	74 (92.5)	0.987 (0.304–3.199)	0.982	0.379 (0.047–3.066)	0.363
Functional outcome at 90-days							
mRS 0-1	79 (49.1)	47 (58)	32 (40)	0.482 (0.257–0.904)	0.023	2.566 (0.597–11.031	0.205
mRS 0-2	92 (57.1)	53 (65.4)	39 (48.8)	0.503 (0.267–0.947)	0.033	4.547 (0.714–28.942)	0.109
mRS 6	34 (21.1)	19 (23.5)	15 (18.8)	0.753 (0.352–1.612)	0.465	0.001 (0.000–0.188)	**0.009**

*sICH, symptomatic intracranial hemorrhage; aICH, asymptomatic intracranial hemorrhage; mTICI, modified treatment in cerebral infarction; mRS, modified rankin score; OR, odds ratio; HR, hazard ratio*.

*adjusted for SBP, NIHSS, atrial fibrillation, smoking, MCA M23 segment, VA, BA, PCA, antiplatelet, Intravenous thrombolysis, general anesthesia, MT stent retrieval, MT aspiration, intra-arterial thrombolysis, balloon angioplasty and retrieval times. Bold values indicates statistical significance*.

**Table 4:** Safety and efficacy outcomes grouped by tirofiban in LAA patients stratified according to minor (NIHSS 0–5)and major (NIHSS > 5) stroke.

**Minor (NIHSS 0**–**5) stroke**							
**Variables**	**Total (*****n*** **= 113)**	**Non-tirofiban (*****n*** **= 75)**	**Tirofiban (*****n*** **= 38)**	**OR/HR**	***P*****-value**	**adjusted OR/HR**	***P*****-value**
Safety outcome							
sICH	2 (1.8)	1 (1.3)	1 (2.6)	2 (0.122–32.881)	0.628	0 (0)	0.993
Recanalization							
mTICI 2b/3	103 (91.2)	70 (93.3)	33 (86.8)	0.471 (0.128–1.742)	0.259	0.095 (0.008–1.070)	0.057
Functional outcome at 90-days							
mRS 0-1	87 (77)	63 (84)	24 (63.2)	0.327 (0.132–0.806)	**0.015**	0.466 (0.122–1.785)	0.265
mRS 0-2	99 (87.6)	69 (92)	30 (78.9)	0.326 (0.104–1.022)	**0.054**	0.551 (0.1–3.034)	0.494
mRS 6	4 (3.5)	2 (2.7)	2 (5.3)	2.028 (0.274–14.986)	0.488	7.76 × 10^3^(0)	0.999
**Major (NIHSS** ***>*** **5) stroke**							
**Variables**	**Total (*****n*** **= 536)**	**Non-tirofiban (*****n*** **= 330)**	**Tirofiban (*****n*** **= 206)**	**OR/HR**	***P*****-value**	**adjusted OR/HR**	***P*****-value**
Safety outcome							
sICH	25 (4.7)	15 (4.5)	10 (4.9)	1.071 (0.472–2.432)	0.869	0.569 (0.071–4.584)	0.596
Recanalization							
mTICI 2b/3	502 (93.7)	307 (93)	195 (94.7)	1.328 (0.633–2.785)	0.453	0.784 (0.183–23.360)	0.743
Functional outcome at 90-days							
mRS 0-1	208 (38.8)	119 (36.1)	89 (43.2)	1.349 (0.945–1.925)	0.099	2.361 (1.326–4.202)	**0.004**
mRS 0-2	265 (49.4)	158 (47.9)	107 (51.9)	1.177 (0.83–1.667)	0.36	1.944 (1.090–3.469)	**0.024**
mRS 6	83 (15.5)	57 (17.3)	26 (12.6)	0.692 (0.419–1.141	0.149	0.252 (0.103–0.621)	**0.003**

*sICH, symptomatic intracranial hemorrhage; aICH, asymptomatic intracranial hemorrhage; mTICI, modified treatment in cerebral infarction; mRS, modified rankin score; OR, odds ratio; HR, hazard ratio*.

*adjusted for SBP, NIHSS, atrial fibrillation, smoking, anterior circulation, posterior circulation, MCA M23 segment, VA, BA, PCA, antiplatelet, Intravenous thrombolysis, general anesthesia, MT stent retrieval, MT aspiration, intra-arterial thrombolysis, balloon angioplasty and retrieval times. Bold values indicates statistical significance*.

**Figure 2:**
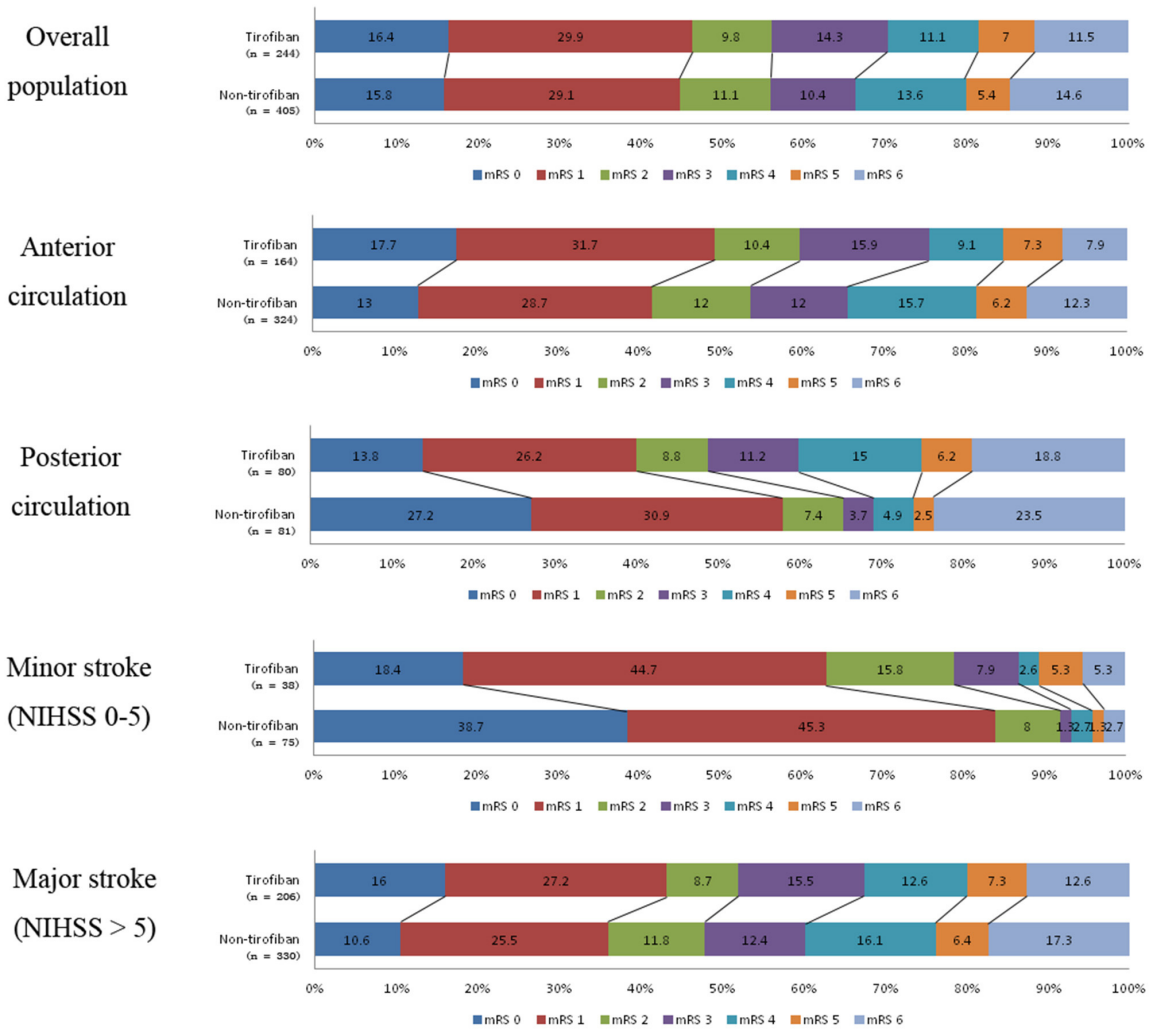
Distribution of mRS scores at 3-month follow-up between tirofiban and non-tirofiban in different stratification.

At 90 day follow-ups, excellent outcome (mRS0-1) and functional independence (mRS0-2) could be achieved in 295 (45.5%) and 182 (44.9%) patients, respectively. However, 87 (13.4%) patients had died (mRS 6) by the three-month follow-up (Table 2, Figure 2). A slightly higher rate of superior clinical outcomes and a lower risk of mortality were found in patients who received tirofiban. Moreover, tirofiban was associated with excellent outcomes and functional independence after adjusting for several potential confounders (adjusted OR, 1.819; 95%CI, 1.064–3.110; *P* = *0.029* and OR, 1.849; 95%CI, 1.065–3.212; *P* = *0.029*, respectively). Further analysis showed a strong association of tirofiban with favorable functional outcomes in the anterior circulation (adjusted OR 2.163; 95%CI, 1.130–4.140; *P* = *0.02*) and NIHSS > 5 (adjusted OR 2.361; 95% CI, 1.326–4.202; *P* = *0.004*). Furthermore, tirofiban was significantly correlated with a lower risk of mortality (adjusted HR 0.2; 95% CI, 0.079–0.507; *P* = *0.001*) even after adjusting for potential factors. This strong association was significantly demonstrated in the anterior circulation (adjusted OR 0.159; 95% CI, 0.042–0.599; *P* = *0.007*), posterior circulation (adjusted OR 0.001; 95% CI, 0.000–0.188; *P* = *0.009*), and NIHSS > 5 (adjusted OR 0.252; 95% CI, 0.103–0.621; *P* = *0.003*).

## Discussion

The present study showed that rescue tirofiban offers a safe outcome for the risk of sICH in AIS patients with LAS who received EVT. In the LAS population, rescue tirofiban showed superior clinical outcomes in patients with an AC stroke and NIHSS > 5. Rescue tirofiban may lower the mortality risk in these stratified patients as well as those with a PC stroke.

The clinical benefit of tirofiban remains controversial in AIS patients who received recanalization therapy. Previous studies have reported the feasibility and effectiveness of tirofiban, and suggested tirofiban use in failed mechanical thrombectomy (15–17). In contrast, another study reported no clinical benefit and also highlighted safety concerns of tirofiban (19). These conflicting results might be attributed to the small sample size, various treatment strategies, and uncontrolled study design in these preliminary studies. Thus, special caution is needed when interpreting these results. However, the majority of these studies shared similar indications that tirofiban is more beneficial for LAA patients. Moreover, a recent meta-analysis indicated that tirofiban use is safe and appears to be effective in treating AIS patients (11–14). Since we compared tirofiban and non-tirofiban use only in patients with LAA, the clinical benefit of rescue tirofiban was more significant in this study. Interestingly, our results demonstrated that patients with an AC stroke and a major stroke received more clinical benefit either through functional outcome or mortality risk from rescue tirofiban, while no significant clinical benefit was found in patients with PC stroke and minor stroke. Despite no functional benefit in those with a PC stroke, rescue tirofiban was advantageous in lowering the mortality rate in the study.

This study was in agreement with previous findings that showed rescue tirofiban did not affect recanalization (1, 21). However, the clinical benefit of rescue tirofiban in LAA patients is that it prevents subsequent ischemic events and the mechanisms have been well-described. Tirofiban has anti-inflammatory effects and may stabilize inflamed stenotic lesions and maintain blood flow, which is helpful in preventing ischemic events caused by inflammation and platelet aggregation (22). In addition, this rescue therapy might benefit cases with stent retrieval times > 3, which are prone to vascular endothelial injury or instant re-occlusion (21, 23). Moreover, it is recommended to use tirofiban in patients with no history of anti-platelet, as it has more significant dose-dependent blockade effects on platelet aggregation and thrombosis (24, 25). Tirofiban is a highly selective platelet antagonist that can block fibrinogen, and its mechanical effect is usually maintained for 20 min after administration (26).

The current study showed that not all LAA patients may receive clinical benefit from rescue tirofiban, including those with a PC stroke or a minor stroke. Accordingly, we assumed that the dosage of tirofiban may account for the clinical benefits in different stratified populations. Based on previously reported medication regimes of tirofiban in AIS patients undergoing EVT, we adopted an intra-arterial administration of < 1 mg and an intravenous infusion of 0.1 μg/kg/min for 12–24 h in patients refractory to recanalization (10). The present study demonstrated that this low- dose rescue tirofiban was effective in cases of AC stroke and major stroke. Nevertheless, since tirofiban was administered within the dosage range in our study, it might have different treatment effects in AIS patients under certain circumstances and may confound the therapeutic effects at a particular dose. Thus, further study with dose-escalation methods is needed for verification. In addition, the present study demonstrated that the use of tirofiban had more favorable outcome in anterior circulation strokes than in posterior circulation strokes. The possible postulated mechanisms attributed to this result may be due to the pathologic mechanisms of stroke and the fact that treatment modalities were significantly different in anterior and posterior circulation, which affect their clinical outcome (27). Posterior circulation stroke patients often presented severe preoperative symptoms and required longer emergency procedures, leading to poor neurological function recovery (27). In addition, the goal for rescue tirofiban is mainly to maintain blood flow and prevent acute occlusion. However, this issue remains uncertain and needs further large prospective trials or randomized controlled trials for verification.

This study had several limitations. First, an uneven proportion between the tirofiban and non-tirofiban groups may cause a bias. Second, the EVT and several other rescue therapies were undertaken at individual discretion, which might affect the treatment results. However, the indications triggering the use of rescue tirofiban were in accordance with standard clinical practice. Third, as the patients enrolled in this study were from China, the results cannot be generalized to the global population. Nonetheless, a strength of the current study was the relatively large sample size compared to previous studies. However, further randomized controlled trials are needed for verification.

## Conclusions

Low-dose rescue tirofiban is safe in AIS patients with LAA, may provide clinical benefit to those with AC stroke or major stroke, and had a tendency to reduce the risk of mortality. However, large cohort or randomized controlled trials with dose-escalation are urgently needed for further verification.

## Data Availability Statement

The raw data supporting the conclusions of this article will be made available by the authors, without undue reservation.

## Ethics Statement

The studies involving human participants were reviewed and approved by ethics committee of Beijing Tiantan Hospital. The patients/participants provided their written informed consent to participate in this study.

## Author Contributions

ZM, YlW, and YjW conceived and led the project. DM, FG and NM supervised and performed quality control for the study. AW performed statistical analysis, XH and R acquired the data and co-wrote the manuscript with input from all co-authors. All authors contributed to the article and approved the submitted version.

## Conflict of Interest

The authors declare that the research was conducted in the absence of any commercial or financial relationships that could be construed as a potential conflict of interest.
